# Dynamic variations in the peripheral blood lymphocyte subgroups of patients with 2009 pandemic H1N1 swine-origin influenza A virus infection

**DOI:** 10.1186/1743-422X-8-215

**Published:** 2011-05-10

**Authors:** Xichao Guo, Yu Chen, Xuefen Li, Haishen Kong, Shigui Yang, Bo Ye, Dawei Cui, Wei Wu, Lanjuan Li

**Affiliations:** 1 State Key Laboratory for Diagnosis and Treatment of Infectious Diseases, First Affiliated Hospital, School of Medicine, Zhejiang University, Hangzhou, 310003, PR China; 2 Department of Laboratory Medicines, First Affiliated Hospital, School of Medicine, Zhejiang University, Hangzhou, 310003, PR China

## Abstract

**Background:**

Novel Influenza A (H1N1) is an acute respiratory infectious disease. Animal experiments indicated that when H1N1 virus infected early hosts, it showed strong CD4^+^, CD8^+^, and CD4^+^CD25^+ ^T cell reactions. The aim of this study was to investigate the dynamic fluctuations of the peripheral blood lymphocyte subgroups in patients infected with H1N1 swine-origin influenza A virus (S-OIV).

**Methods:**

The frequency of T cells, B cells, natural killer (NK) cells, and regulatory T cells (Treg) in 36 severe H1N1 and 40 moderate H1N1 patients were detected at different periods by flow cytometry. In parallel, serum cytokines were detected by enzyme-linked immunosorbent assay and C-reactive protein (CRP) was analyzed through an image-type automatic biochemical analyzer. In addition, 20 healthy volunteers, who were not infected with 2009 H1N1 virus, were selected as controls.

**Results:**

The frequency of NK cells were decreased in all cases and CD19^+ ^B cells were increased in severe cases than those of the controls. At 1-2d from onset, the frequency of CD4^+ ^and CD4^+^CD25^+ ^T cells in moderate cases was higher than in the severe cases. Serum cytokines, specifically IL-2, IL-4, IL-6, IL-10, and IFN-γ exhibited no significant change both in the moderate and the severe cases during the whole monitoring process. In the early stage of the disease, serum CRP levels in the severe and moderate groups were significantly higher than that in the control group.

**Conclusions:**

Patients showed different lymphocyte subgroup distributions between mild and severe cases, which might affect the incidence and development of 2009 H1N1.

## Background

The latest public data from the Chinese Ministry of Health showed 120,940 confirmed H1N1 cases and 659 deaths were reported from the Chinese mainland as of January 2, 2010. Data analysis suggests that patients with chronic diseases, obese patients, and pregnant women are more liable to develop severe H1N1 influenza. After initial infection by the virus, the host innate immune system, as a first line of defense, takes protective measures earlier than the adaptive immune system to avoid further viral invasion or replication [1]. Animal experiments indicated that when the swine-origin influenza A virus (S-OIV) infected early hosts, it elicited strong CD4^+^, CD8^+^, and CD4^+^CD25^+ ^T cell reactions [2]. CD4^+ ^and CD8^+ ^T cells are related to viral immunity and the lack of these cells would lead to a delay in viral clearance and an increase in mortality [3-5]. On the other hand, CD4^+^CD25^+ ^cells are T cell subsets with an immune inhibition function and could play an important regulatory role in a variety of infectious diseases [6-8]. These complex molecular and cellular mechanisms are helpful in controlling and eliminating the acute stage of viral infection. Although successfully eliminating the virus is essential, the response of virus-specific T cells to the hosts could also result in tissue damage and autoimmune responses. Therefore, monitoring the variation of cellular immune functions in H1N1 patients have important clinical significance. The aim of this study is to analyze the dynamic fluctuations of T cells, B cells, natural killer (NK) cells, and regulatory T cells in patients infected with novel influenza H1N1, as well as serum cytokines and C-reactive protein (CRP).

## Methods

### Enrollment of study subjects

Up to 76 H1N1 outpatients and inpatients were chosen from August to December 2009 in the Department of Infectious Diseases, First Affiliated Hospital, School of Medicine, Zhejiang University. Among these, 36 cases were severe and 40 cases were moderate (Table 1). In addition, 20 healthy volunteers who were not infected with 2009 H1N1 virus, were selected as controls. There were no statistical differences between their ages and genders compared with the H1N1 patients.

**Table 1:** Basic characteristic of patients with severe H1N1and moderate H1N1

Variable	severe H1N1 (*n *= 36)	moderate H1N1 (*n *= 40)	*P*-value
Age, mean ± SD, years*	40.1 ±18.6	26.1 ± 8.0	0.006
Male/Female distribution, *N *(%) ^▲^	20/25(55%/45%)	20/20(50%/50%)	0.653
Prodromal symptoms, *N *(%) ^▲^			
Fever(T > 38.5)	28(78)	16(40)	0.001
Cough	26(72)	28(70)	1.000
Dyspnea	13(36)	0	<0.001
Gastrointestinal	4(11)	3(7)	0.702
Myalgia	23(64)	10(25)	0.001
Co-morbidities, *N *(%) ^▲^			
COPD	12(33)	0	<0.001
Coronary artery disease	4(11)	0	<0.001
Diabetes mellitus	5(14)	2(5)	0.246
Hypertension	5(14)	2(5)	0.246
Pregnancy	4(11)	1(2.5)	0.131
Others (Hypothyroidism, Immunosuppression, Transplant)	3(8)	0	0.102
Changes in lung imaging examination, *N *(%) ^▲^	20(55)	3(7)	<0.001
Patients using Oseltamivir, *N *(%) ^▲^	36(100)	40(100)	1.000
Oseltamivir treatment time, mean ± SD, (days)*	8.55 ± 3.39	3.60 ± 1.00	<0.001
Patients using combined glucocorticoid, *N *(%) ^▲^	19(53)	0	<0.001
The number of people using the ventilato, *N *(%) ^▲^	5(14)	0	<0.001
Average stay, mean ± SD, days*	13.71 ± 4.77	3.37 ± 1.06	<0.001
Death toll, *N *(%) ^▲^	1(3)	0	0.474

Note: SD = standard deviation; COPD = chronic obstructive pulmonary disease. * Date are reported as mean ± SD; ^▲ ^Date are expressed as no. (%).

The diagnostic criteria for H1N1 were based on the Influenza A H1N1 Flu Clinic Program (pilot version of the second edition, 2009) released by the Chinese Ministry of Health in July 2009. Its main content includes the following:

I. Confirmation standard of Influenza A (H1N1) cases: Patients are considered to have contracted the virus if they present with influenza-like clinical manifestations and had one or more of the following laboratory test results: (1) Positive for S-OIV nucleic acid (through real-time RT-PCR and RT-PCR); (2) S-OIV is isolated and attained; and (3) Specific neutralizing antibody titers for serum S-OIV are increased four times or more.

II. Severe cases: When confirmed or suspected cases manifest one of the following signs, they are considered as severe: (1) Complicated pneumonia with or without hypoxemia and respiratory failure; (2) toxic shock; and (3) multiple organ dysfunction syndrome or multiple organ failure.

The study protocol was approved by the Ethics Review Committee of the First Affiliated Hospital, School of Medicine, Zhejiang University. All subjects understood the procedures and consented participate in the study.

### Sample collection and analysis

On day 1, 2, 3, 5, 7, 9, 11, 14, and 21, 2 mL of whole blood (EDTA-K2 anticoagulant) and 1.5 mL of serum were collected from H1N1 patients for the detection of lymphocyte subgroups, cytokines, and CRP. The same sample collection was carried out in the control group.

Afterwards, 100 μL of anticoagulant was added into all flow tubes and 20 μL of CD3-PC5, CD4-FITC, CD8-PE, CD3-FITC-CD(16+56)-PE, CD19-FITC, and CD25-PE mouse anti-human fluorescence monoclonal antibodies (BD Bioscience) was added. After mixing, the samples were incubated for 15 min away from light at room temperature. Red blood cell lysis and cell fixation using Coulter QPREP specimen processing instrument (Beckman Coulter Inc., USA) was conducted, and the cells more than 1 × 10^4 ^were counted through a flow cytometer (Coulter Cytomics FC 500, Beckman Coulter). The results were expressed as the percentages of CD4^+^CD25^+^, CD4^+^, and CD8^+ ^T cells, as well as those of CD19^+ ^B cells and NK (marked as CD3^-^CD16^+^CD56^+^) cells found positive for the antigen marker in the total lymphocytes population. The data were collected and analyzed using the EXPO32 software (Beckman Coulter Inc., USA).

Serum CRP detection was conducted using the image-type automatic biochemical analyzer (Beckman Coulter Inc., USA). The kit was also provided by the same company. Serum cytokines, namely IL-2, IL-4, IL-6, IL-10, and IFN-γ were tested using an enzyme-linked immunosorbent assay (ELISA; Ex-cell). The experimental methods were in accordance with the instructions of the manufacturer in the reagent kit.

### Statistical analysis

SPSS 13.0 statistical package was used for data processing. Measurement data were indicated as mean ± standard deviation or percentages. Comparisons among groups were carried out using one-way ANOVA or χ^2 ^test. Differences with P < 0.05 were considered statistically significant. Correlation analysis among the detection indicators were conducted using Spearman's rank correlation analysis.

## Results

### Demographic characteristics and clinical features of H1N1

Up to 76 patients with confirmed laboratory diagnoses of influenza were enrolled in this study. The baseline characteristics of the patients are summarized in Table 1. The samples were predominantly composed of full-grown adults with S-OVI infection.

As shown in Table 1, the patients with severe H1N1 were significantly older than those that presented with moderate H1N1 (mean age, 40.1 years vs. 26.1 years; P < 0.01). The mean duration of symptoms before presentation was 4 days, with fever, cough, and myalgia as the most common in both groups. However, the patients with severe H1N1 had more prodromal symptoms (e.g., fever, dyspnea, and myalgia) and co-morbidities [e.g., chronic obstructive pulmonary disease (COPD) and coronary artery disease] than the patients with moderate H1N1 (p < 0.01). Although a relationship with pregnancy was more common in the severe H1N1 group (four patients vs. one patient with moderate H1N1), this difference was not statistically significant (Table 1). Similarly, there was no difference in the other preexisting diseases such as diabetes mellitus and hypertension between the two groups.

Chest x-rays were taken from all patients, and abnormalities were detected in 23 patients, 3 with moderate H1N1 and 20 with severe H1N1. Ten patients presented with radiological abnormalities attributable to chronic lung conditions without evidence of concurrent pneumonia.

Up to 15 patients with severe H1N1 were admitted to the intensive care unit (ICU) with preexisting diseases, such as COPD. All patients were treated with Oseltamivir (Tamiflu) and 53% of the severe H1N1 patients were additionally treated with glucocorticoids (19/36). The duration of Oseltamivir treatment was based on the Pandemic Influenza A (H1N1) 2009 Clinical Guidelines (Second Edition, 2009). However, the subjects were not given immunomodulators. Treatment response did not differ significantly between the two groups and the mean duration of hospital confinement was 13.7 days (range, 0-51 days) and 3.3 days (range, 0-11 days) for the severe and moderate patients, respectively. Overall, only one severe H1N1 patient died during the course of the study. No patient dropped out and all completed the 21-week follow-up.

### Peripheral blood lymphocyte subgroups changes

The frequency of peripheral blood CD19 ^+ ^B cell count of patients with severe H1N1 gradually increased within the first 3-14 d of treatment. The NK cell count showed a gradual decline, whereas the T cell subtypes (CD4^+^CD25^+^, CD4^+^, and CD8^+ ^T cells) showed no significant changes. The frequency of peripheral CD4^+^T and CD4^+^CD25^+^Treg cells of patients with moderate H1N1 were significantly higher than in patients with severe H1N1 within the first 1-2 d of treatment. The NK cell counts of patients with moderate H1N1 were similar to that of patients with severe H1N1, which demonstrated a gradual decline, whereas the frequency of CD19^+ ^B and CD8^+ ^T cells showed no significant changes.

**Figure 1:**
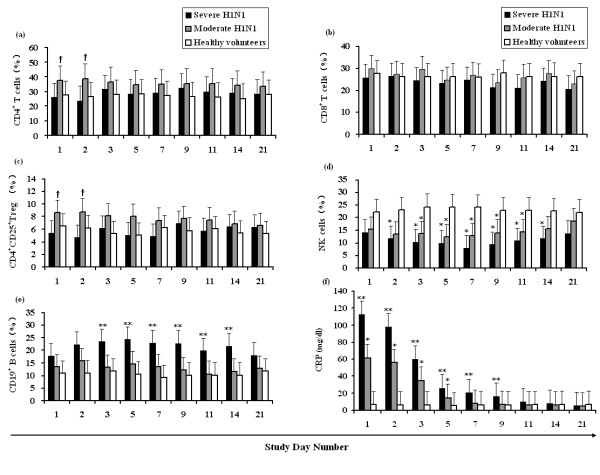
**Analysis of the dynamic changes in the lymphocyte subgroup and C-reactive protein (CRP) in the peripheral blood of patients with Influenza A (H1N1) 2009**. CD4^+ ^T, CD8^+ ^T, CD4^+^CD25^+ ^Treg, NK, and CD19^+ ^B cells are presented as a percentage of total peripheral blood leukocytes (PBLs) in severe H1N1 (*n *= 36) patients, moderate H1N1 (*n *= 40) patients, and healthy volunteers (*n *= 20). **†**: denotes statistically significant differences compared with severe H1N1; *: denotes statistically significant differences compared with the healthy volunteers; **: denotes statistically significant differences compared with the moderate H1N1 patients and with the healthy volunteers.

The frequency of the different lymphocyte subsets at different periods were statistically compared and the results show that the frequency of peripheral CD4^+ ^and CD4^+^CD25^+ ^T cells in the moderate H1N1 patients were higher than that of the severe H1N1 patients within the first 1-2 d (Figures 1a and 1c; P < 0.05). The CD19^+ ^B cell counts of the severe H1N1 patients were significantly higher than those of the moderate H1N1 patients and the control group for the same period (Figure 1e), whereas the NK cell counts were significantly less than that of the control group (Figure 1d; P < 0.05). The frequency of CD8^+ ^T cells had no significant difference (P > 0.05; Figure 1b) among the three groups.

### Behavior of the serum CRP responses against a sustained treatment

In the first 1-5 d of study, the serum CRP levels of both the patients with severe H1N1 and the patients with moderate H1N1 were significantly higher than that of the control group (P < 0.01; Figure 1f). Correlation analysis of the CD4^+^, CD8^+ ^T, CD4 ^+^CD25^+^, CD19^+ ^B, and NK cells with CRP was conducted and the results indicate that the various lymphocyte subgroups had no significant correlations with CRP.

### Induction of serum cytokines activity

The serum cytokines of patients, specifically IL-2, IL-4, IL-6, IL-10, and IFN-γ, whether the H1N1 infection was severe or moderate, showed no significant changes during the whole monitoring process (data not shown). The differences of the H1N1 patients with the control group had no statistical significance.

## Discussion

Influenza A (H1N1) is the greatest pandemic threat in 2009 [9]. The clinical course of H1N1 can be severe, particularly for very young patients with complications. The immune response affected by pathological damage to the body caused by S-OVI and clinical prognosis. Studies have shown that the incidence of patients with severe H1N1 was highest in the 15- to 19-year-old age group and the lowest for patients aged 65 or above [10]. Our clinical data also showed that the most severe infections occur in individuals younger than 25 years old or in middle-aged patients older than 55 years old with comorbidities, especially lung disease.

Based on local guidelines, antiviral treatment would be considered for severe or critical cases and high-risk patients infected with the influenza A pandemic (H1N1) virus within 48 h of onset. For patients who presented later than 2 days, the managing physicians were allowed to make decisions regarding antiviral use. Oseltamivir, given to patients older than 12 years, was prescribed according to the standard dosing regimen (75 mg twice daily orally, for 5 days). Dosage adjustment, whenever necessary, were based on the patient's renal function (75 mg daily, if creatinine clearance is <30 mL/min). For children (1-12 years old), dosage adjustments were based on body weight (BW), that is: 30 mg twice daily for children with BW < 15 kg; 45 mg twice daily for BW 15-23 kg; 60 mg twice daily for BW 23-40 kg; and 75 mg twice daily for BW > 40 kg. Accordingly, patients with acute respiratory distress syndrome (ARDS) were treated with high dose glucocorticoids over a short period of time, which has been proven beneficial and safe.

Several lines of experimental evidence suggest that H1N1 infections accompanied by a characteristic impairment of the innate immune response. By monitoring the host functional response, patients with increased risks of developing severe influenza-associated complications could be identified immediately [11,12]. The preliminary data from this study showed that the frequency of CD19^+ ^B cells in patients with severe H1N1 was significantly higher than in the moderate and control groups (Figure 1e), and the frequency of NK cells in the severe and moderate groups were less than that of the control group. In the host innate immune system, NK cells are the major effector cells during acute infection, rapidly killing infected cells in the process. Previous studies have shown that the significant decrease in peripheral NK cell count is mainly because the S-OVI directly infected and killed NK cells, thereby limiting their activity and leading to their apoptosis in vivo [13].

Animal studies have found that CD4^+ ^T, CD8^+ ^T, and CD19^+ ^B cells achieved peak values within 5-6 d after S-OVI infection, whereas the CD4^+^CD25^+ ^Treg cell count reached its peak at 24-48 h after infection [2]. Currently, the use of flow cytometric analysis to measure peripheral blood CD4^+^, CD8^+^, and CD4^+^CD25^+ ^T cells is a simple and common method. Reportedly, the mostly CD4^+^CD25^+ ^T cells were also positive for FoxP3 [14]. In this experiment, the peak values for all lymphocyte subgroups in patients with severe H1N1 could not be detected. At least two mechanisms could account for the lower effector T cell response detected after the S-OVI infection, and the peak response time for the effector T cells possibly missed. In addition, the virus could have also led to the immunotolerance of the T cells [15].

As intercellular signaling molecules, cytokines regulate the immune response, mediate inflammatory responses, and participate in tissue repair. Experiments have shown that only the peak values of IFN-α and IL-6 could respectively be detected within 24 and 30 h in the serum of infected animals, as well as in the plasma of pediatric patients with severe influenza [16,17]. In this experiment, no statistically significant changes were found during the continuous monitoring of serum IL-2, IL-4, IL-6, IL-10, and IFN-γ of patients with severe and moderate H1N1, as well as in the control group. This result might also be related to the immunotolerance of T cells, of which the peak detection might have been missed. The mechanisms responsible for maintaining these relatively constant levels remain unclear.

CRP is an acute phase protein produced by the liver and is a sensitive marker for the inflammatory response. It did not directly related to viral load [17]. However, CRP continuously increased in the initial stages of infection for both the patients with severe and those with moderate H1N1. The CRP of patients with severe H1N1 took longer to return to normal, which might be the result of underlying disease. Each lymphocyte subgroup has made correlatively analyzed with CRP and the results show no significant correlations.

In conclusion, S-OIV could stimulate the cellular immune response. When accompanied by CD19^+ ^B cell increase and a continuous NK cell decline, it could indicate that the body is in a dynamic balance of the anti-infection immunity and autoimmune damage; this is particularly true for patients with severe influenza. Each lymphocyte subgroup in patients with H1N1 plays a more important antiviral role in the early stages of disease, but excessive immune response also leads to the increase and development of infection.

## Competing interests

The authors declare that they have no competing interests.

## Authors' contributions

XG and YC performed the majority of experiments and drafted the manuscript. XL and HK followed-up the patients and involved in editing the manuscript. SY and WW did most of clinical works. BY and DC provided analytical tools. LL was the principal investigator and provides all facilitates to complete this work. All authors read and approved the final manuscript.
